# Acupuncture combined with exercise therapy for Sarcopenia in the elderly: A multicenter randomized controlled study protocol

**DOI:** 10.1016/j.mex.2025.103655

**Published:** 2025-10-06

**Authors:** Yangchi Li, Yu Lu, Wengxiong Li, Feng Yang

**Affiliations:** a Shaanxi University of Chinese Medicine, Xianyang 712046, China; b Affiliated Hospital of Shaanxi University of Chinese Medicine, Xianyang 712000, China; c Shaanxi Key Laboratory of Chronic Musculoskeletal Diseases, Xianyang 712000, China; d Shaanxi University Youth Innovation Team for TCM prevention and treatment of bone and joint diseases research and innovation team, Xianyang 712046, China

**Keywords:** Sarcopenia, Acupuncture, Irisin, Myostatin, Multi-center study, Sham control group

## Abstract

Sarcopenia (SP) is an age-related disorder characterized by progressive loss of muscle strength, mass, and limb dysfunction, severely compromising the quality of life and life safety in older adults. With the global aging population, the incidence of this disease has been escalating, posing a significant public health challenge. Preclinical studies have demonstrated that acupuncture effectively mitigates muscle loss in rats; however, the field currently lacks robust randomized controlled trials (RCTs), marked by limitations such as the absence of sham acupuncture controls, multicenter designs, and standardized outcome metrics. Therefore, this study integrates acupuncture with exercise therapy, incorporating the following design highlights:

A multicenter approach to enhance research reliability;

A control group receiving sham acupuncture combined with exercise to eliminate the placebo effect;

The most comprehensive and validated set of outcome measures to ensure study rigor and expand the conceptual framework for sarcopenia assessment.

This research aims to provide robust evidence for acupuncture as an effective adjuvant therapy for SP. The study has been registered with the International Traditional Medicine Clinical Trials Registry Platform (ITMCTR2024000374).

## Graphical abstract

**Figure:**
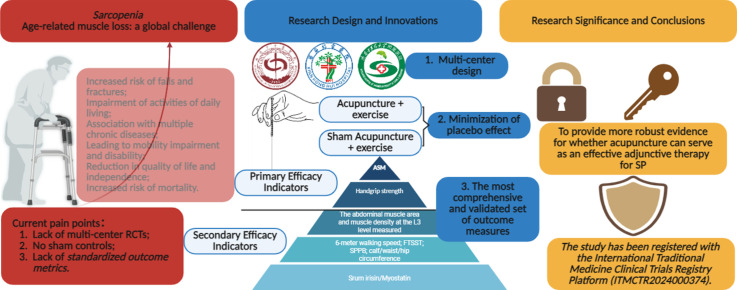

**Abbreviations**
SP: Sarcopenia
RCTs: Randomized Controlled Trials
ASM: Appendicular Skeletal Muscle Mass
SMI: Skeletal Muscle Index
DXA: dual-energy X-ray absorptiometry
QCT: Quantitative Computed Tomography
FTSST: Five-Times-Sit-to-Stand Test
SPPB: Short Physical Performance Battery
IGF-1: Insulin-like Growth Factor-1
TNF-α: Tumor Necrosis Factor-α
IL-6: Interleukin-6
SOD: Superoxide Dismutase
GSH-Px: Glutathione Peroxidase
CRC: Clinical Study Coordinator
ECG: Electrocardiogram
FAS: Full Analysis Set
PPS: Per Protocol Set
SS: Safety Set
ITT: Intention-To-Treat
DMC: Data Monitoring Committee
IEC: Ethics Committee

**Specifications table**

**Table:** 

**Subject area**	Medicine and Dentistry
**More specific subject area**	Traditional Chinese Medicine (TCM) and acupuncture for the treatment of musculoskeletal disorders.
**Name of your protocol**	Acupuncture combined with Exercise Therapy for Sarcopenia in the Elderly: A Multicenter Randomized Controlled Study Protocol.
**Reagents/tools**	Not applicable.
**Experimental design**	This study is a prospective, multi-center, parallel-group randomized controlled trial, which is a superiority intervention study. SP patients who meet the diagnosis and inclusion criteria will be recruited. Patients will be randomly assigned to two groups in a 1:1 ratio: the acupuncture group (receiving acupuncture at selected points combined with exercise therapy) and the sham acupuncture group (receiving sham acupuncture combined with exercise therapy). Both groups will be treated for 60 days and followed up for 90 days. Various efficacy indicators and safety indicators of the patients will be recorded before treatment, at 30 days of treatment, at 60 days of treatment, 60 days after the end of treatment, and 90 days after the end of treatment. After randomization, the Clinical Study Coordinator(CRC) will schedule treatment sessions. All recruitment procedures would be documented in the log file.This trial protocol uses the Standard Protocol Items :Recommendations for Interventional Trials (SPIRIT) reporting guidance Fig. 1 shows a flow chart of the study design.
**Trial registration**	The study has been registered with the International Traditional Medicine Clinical Trials Registry Platform (ITMCTR2024000374).
**Ethics**	The study protocol has been reviewed and obtained ethics approval from the IEC of The Affiliated Hospital of Shaanxi University of Chinese Medicine (SZFYIEC-PJ-2024No.[156]). All subjects involved in this study will sign the informed consent form. And our informed consent form contains the subjects' permission for all the data that we will collect and use. Further analysis of biological samples is not applicable to this study.
**Value of the Protocol**	1. SP severely compromises quality of life and survival in older adults, with its incidence escalating alongside global aging. However, mainstream treatments for SP are associated with limitations: exercise therapy alone suffers from low adherence, nutritional support carries the risk of exacerbating renal burden, and hormone therapy exhibits significant side effects. Thus, there is an urgent need to explore effective adjunctive and alternative therapies for SP. 2. Previous clinical studies on acupuncture for SP have been plagued by flaws such as the absence of multicenter designs and lack of sham controls. Therefore, this study will employ a multicenter RCT to enhance reliability, complemented by a sham acupuncture control group to minimize the placebo effect. 3. Previous research in this field has suffered from inconsistent and incomplete efficacy endpoints. Thus, this study introduces a comprehensive set of primary and secondary endpoints, encompassing muscle strength, muscle mass, physical function, and muscle-related serum biomarkers.

## Background

Sarcopenia(SP) is mainly characterized by the progressive loss of skeletal muscle mass and function [[1], [2], [3]]. As a disease closely associated with aging, the prevalence of SP has been increasing year by year along with the intensifying global aging trend, thus emerging as a global public health concern and a cutting-edge research topic. Worldwide, in the population of community - dwelling elderly individuals, the prevalence of SP spans from 10 % to 40 % [4]. The decreased muscle strength, reduced muscle mass, and limb dysfunction manifested by SP severely impair the quality of life and life safety of the elderly. Patients with SP have a 2 - 3 times higher risk of falling compared to the normal population [[5], [6], [7]]. The pathogenesis of SP involves multiple factors. Various chronic diseases, malnutrition, and lack of exercise that occur during the aging process of the human body [8,9], as well as inflammatory responses, oxidative stress, mitochondrial dysfunction, and neuromuscular junction degeneration at the molecular level, are all important contributors to SP [[10], [11], [12]].

Acupuncture represents an effective approach for the treatment of SP [13]. Acupuncture, based on the theory of Chinese medicine meridians, stimulates specific acupoints to regulate the circulation of qi and blood, the functions of zang-fu organs, and the waxing and waning of qi and blood in the meridians, thereby achieving the goals of strengthening the healthy qi, expelling pathogenic factors, and balancing yin and yang [5]. From the perspective of modern medicine, the mechanisms underlying acupuncture treatment for SP are multifaceted. It may regulate the neuroendocrine system, promoting the secretion of hormones related to muscle growth and repair, such as growth hormone and insulin-like growth factor-1 (IGF-1) [14]. This, in turn, stimulates muscle protein synthesis, inhibits muscle protein breakdown, and ultimately increases muscle mass and strength. Meanwhile, the anti-inflammatory and antioxidant effects of acupuncture contribute to alleviating inflammatory responses and oxidative stress damage in muscle tissues, protecting muscle fibers, improving the muscle microenvironment, and facilitating muscle repair and regeneration [5,10]. Additionally, acupuncture can modulate the function of the nervous system, enhancing neuromuscular conduction, increasing muscle excitability and coordination, and further elevating muscle strength and motor function. Some basic research and clinical observations have provided evidence to support acupuncture treatment for SP [5,14]. For instance, animal experiments have revealed that acupuncture intervention can significantly improve muscle mass, strength, and exercise capacity in animal models of SP [10]. The underlying mechanisms are associated with regulating muscle-related gene expression, promoting protein synthesis, and suppressing inflammatory responses. In clinical practice, several studies have also reported that acupuncture exhibits certain efficacy in improving SP that occurs concurrently with chronic diseases (such as chronic liver disease, chronic kidney disease, heart failure, etc.), manifested as enhanced muscle strength, improved limb function, and elevated quality of life [15,16].

However, there are relatively few RCTs in this area. Almost none of the existing RCTs have involved sham controls or multi-center studies to enhance reliability. The lack of unified observation indicators also poses challenges to sensitivity, specificity, and reproducibility [[17], [18], [19], [20], [21], [22], [23], [24], [25]]. Given the high prevalence and severe consequences of SP, as well as the potential multi-target action mechanisms and promising clinical application prospects of acupuncture in treating this disease, this protocol has been meticulously designed to observe the efficacy and safety of acupuncture combined with exercise therapy for SP, aiming to provide more scientific and reliable evidence for acupuncture as a reliable complementary therapy for SP.

## Description of protocol

1. Objectives:

This study is a multi-center, randomized, controlled clinical trial, aiming to observe the efficacy and safety of acupuncture combined with exercise therapy for SP, and to provide reliable evidence for acupuncture as a trustworthy complementary therapy in this field.

2. Methods:

### Trial design

**Fig. 1:**
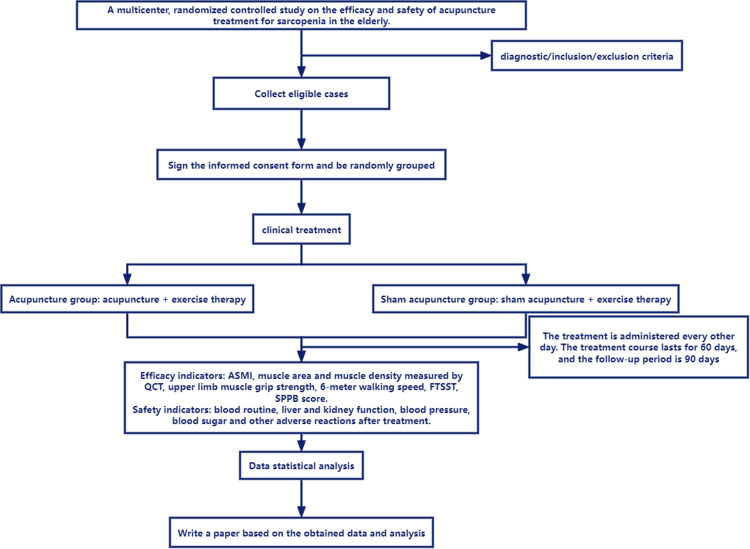
Flow chart of the study design.

This study is a prospective, multi-center, parallel-group randomized controlled trial, which is a superiority intervention study. SP patients who meet the diagnosis and inclusion criteria will be recruited. Patients will be randomly assigned to two groups in a 1:1 ratio: the acupuncture group (receiving acupuncture at selected points combined with exercise therapy) and the sham acupuncture group (receiving sham acupuncture combined with exercise therapy). Both groups will be treated for 60 days and followed up for 90 days. Various efficacy indicators and safety indicators of the patients will be recorded before treatment, at 30 days of treatment, at 60 days of treatment, 60 days after the end of treatment, and 90 days after the end of treatment. After randomization, the Clinical Study Coordinator(CRC) will schedule treatment sessions. All recruitment procedures would be documented in the log file.This trial protocol uses the Standard Protocol Items :Recommendations for Interventional Trials (SPIRIT) reporting guidance [26]. Fig. 1 shows a flow chart of the study design.

### Study setting

The study sites include three tertiary hospitals in Shaanxi, China: (1) The Affiliated Hospital of Shaanxi University of Chinese Medicine, (2) Xi'an Honghui Hospital, and (3) Xi'an Hospital of Traditional Chinese Medicine. The reason why these three hospitals are selected to conduct the clinical trial is that they are equipped with comprehensive examination and treatment facilities. Enrolled patients will fill out questionnaires, undergo examinations, and receive treatment in these three hospitals. Considering that elderly patients with SP may have mobility problems, we plan to provide pick-up and drop-off services for the subjects, subject to the approval of the ethics committee. This is intended to show our concern for the patients and reduce the dropout rate.

### Eligibility criteria

#### Diagnostic criteria

In this study, the diagnostic criteria were formulated with reference to Sarcopenia in Asia: consensus report of the Asian Working Group for sarcopenia, 2019 [1]

(1) Preliminary screening: SARC—CalF questionnaire: a SARC—CalF questionnaire≧11 is classified as a positive SP screen;
(2) Muscle power: Handgrip strength <28 kg for men and <18 kg for women;
(3) Muscle function: Utilizing 6-meter walking speed and FTSST, 6-meter walking speed <1.0 m/s and FTSST >12 s. *Or sppb*≦9 points;
(4) Muscle mass: DXA: ASM, Males <7.0 kg/m^2^, females <5.4 kg/m^2^.

The above item (1) is the case finding session, and (1)(2)(4) are performed on the patient when the SARC—CalF questionnaire is screened positive.

Diagnosis of SP: Decreased skeletal muscle content (positive for item (4))+decreased muscle strength (positive for item (2)) or decreased trunk function (positive for item (3));

#### Inclusion criteria

(1) Patients who meet the aforementioned diagnostic criteria. (2) Patients aged between 60 and 80 years old, regardless of gender. (3) Those who have agreed to participate in this study and have signed the informed consent form.

#### Exclusion criteria

(1) Individuals with severe primary diseases of vital body organs (such as the heart, kidney, liver, etc.) or important systems; (2) Those with limb diseases that prevent movement (e.g., fractures, cerebral infarction, cerebral hemorrhage, etc.) or a combination of diseases that affect body weight (e.g., long-term hormone users); (3) People with a certain degree of limitation in their ability to perform activities of daily living; (4) Persons with electronic devices or metal objects implanted in their bodies (e.g., pacemakers or artificial joints); (5) Individuals with severe psychiatric mental illness or cognitive dysfunction; (6) Those with underlying diseases (hypertension, diabetes, coronary heart disease, etc.) and whose underlying diseases are not in a stable stage; (7) Exclude patients with reduced muscle mass or muscle strength due to other causes (myasthenia gravis, spinal cord injury, diabetes, COPD, etc.); (8) Long-term bedridden patients due to post-stroke, spinal cord injury, physical disability, etc. are excluded; (9) Patients with other muscle diseases such as amyotrophic lateral sclerosis, myotonic dystrophy, etc. that lead to decreased muscle mass or muscle strength are excluded.

**Fig. 2:**
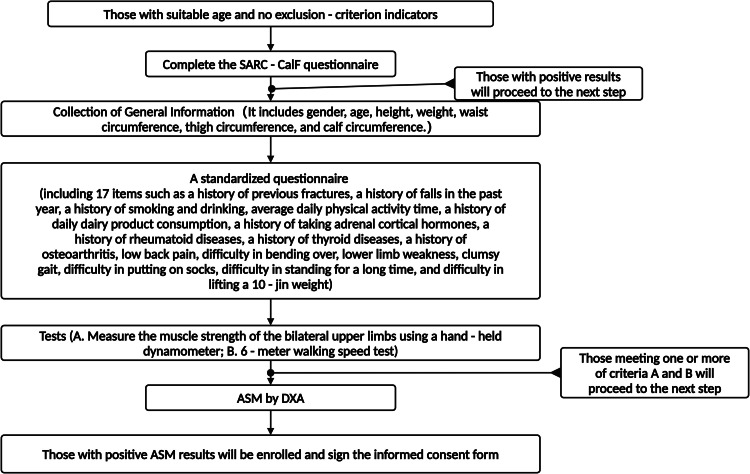
Flowchart of enrollment.

Fig. 2 shows the enrollment process.

### Sample size

Regarding the design of the sample size calculation formula, we referred to the 8th edition of "Epidemiology" published by the People's Health Publishing House [27]. The formula is presented as follows: *N* = 2(Z₁₋α/₂ + Zβ)²σ²/d². In this formula, Z₁₋α/₂ represents the value corresponding to the first type of error probability, and Zβ denotes the value for the second type of error probability. σ is the standard deviation estimated through literature study [[17], [18], [19], [20], [21], [22], [23], [24], [25]], with σ=0.80 kg/m². d is the difference between the means of the two groups as estimated through literature study [[17], [18], [19], [20], [21], [22], [23], [24], [25]]. The mean value was 6.76 kg/m² in the acupuncture group and 6.08 kg/m² in the sham acupuncture group, thus *d* = 0.68 kg/m². Substituting the above data into the formula yields a sample size of 36 cases per group. Considering a 10 % dropout rate, the number of cases to be included in each group should be no <40. Since a 1:1 ratio is employed for both groups, 80 cases are needed at each center, totaling 240 cases.

### Assignment of interventions: allocation

#### Sequence generation

In accordance with central randomization and a completely randomized grouping method, 240 three-digit random numbers were generated by SPSS statistical software. Subsequently, each three-digit number was recorded as a random number corresponding to a patient. The patients were numbered from 001 to 240 in the order of admission, corresponding to the random numbers. Then, the 240 random numbers were sorted from smallest to largest. (The data were consistent when numbered sequentially.) It was stipulated that patients with serial numbers 001 to 120 belonged to the acupuncture group, and those with serial numbers 121 to 240 were in the control group.

#### Concealment mechanism

The computerized randomization program will not release the random codes until the patients are enrolled in the study, thus ensuring allocation concealment. Due to the differences in the operational methods between acupuncture and sham acupuncture, it is impossible to blind the researchers. However, the data collection and analysis will be completed by professional statisticians. The subjects and researchers will be unaware of the data, while the statisticians will be unaware of the study groups.

#### Implementation

The principal investigators at each center will recruit subjects. A computerized randomization program will generate an allocation sequence at a ratio of 1:1. An independent researcher will assign the participants to the acupuncture group and the sham acupuncture group.

### Assignment of interventions: Blinding

Due to the characteristics of the intervention study, in the design of the acupuncture intervention, blinding will not be applied to the subjects and researchers regarding the intervention measures. However, the data analysts will be blinded to the allocation of the intervention measures. The result evaluation and statistical analysis will be conducted by independent investigators who are unaware of the patient grouping.

### Interventions

#### Explanation for the choice of comparators

Exercise therapy is currently the main treatment approach for SP. From the perspectives of traditional Chinese medicine theory and modern medical research, acupuncture has the efficacy of promoting muscle growth. Then, will combining acupuncture with other mainstream therapies for SP further enhance the curative effect? Does acupuncture treatment for SP have a placebo effect? What are the specific mechanisms by which acupuncture improves SP? How effective is acupuncture specifically in terms of indicators such as muscle strength, muscle mass, and muscle function? We still lack strong clinical evidence in these aspects. Therefore, we have designed sham acupuncture combined with exercise therapy as the control group.

#### Intervention description

Eligible subjects will be randomly assigned to two parallel groups.

#### Acupuncture group treatment protocol

**Acupuncture:** For the selection of acupoints, bilateral Zusanli (ST36), Taixi (KI3), Sanyinjiao (SP6), Quchi (LI11), and Hegu (LI4) were selected (referring to the "Three-needle Therapy" published by China Medical Science Press [28] and the "Clinical Rehabilitation" published by People's Health Publishing House [29]). The location of these acupuncture points adheres to the Chinese national meridian point standard (GB/T 12,346 - 2021) [30].

**Fig. 3:**
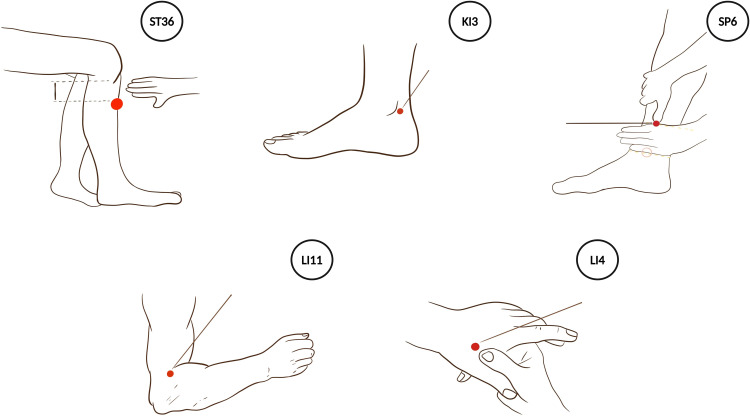
Diagram of acupoint locations.

(1) ST36: It is situated on the lateral side of the lower leg, 3 cun (a traditional Chinese unit of length) distal to Dubi (the lateral patellar fossa), and approximately one fingerbreadth (equivalent to the width of the middle finger) lateral to the anterior border of the tibia;
(2) KI3: Located in the ankle region, it lies in the depression between the tip of the medial malleolus and the Achilles tendon;
(3) SP6: Positioned on the medial side of the lower leg, it is 3 cun proximal to the tip of the medial malleolus and at the posterior border of the medial aspect of the tibia;
(4) LI11: When the elbow is flexed at a right angle, it can be found at the midpoint of the line connecting the lateral end of the elbow crease and the lateral epicondyle of the humerus;
(5) LI4: It is located between the first and second metacarpal bones on the dorsum of the hand, precisely at the midpoint on the radial side of the second metacarpal bone. Fig. 3 depicts the positions of these acupoints;

Operation Method: Patients are required to lie in the supine position with their elbows bent and placed over the chest. The acupuncturist first disinfects their hands thoroughly and then conducts local disinfection of the selected acupoints using 75 % medical alcohol. A 0.30 mm × 40 mm needle is used to puncture the aforementioned points directly. The needling depth ranges from 20 to 30 mm . The tonifying needling technique is adopted for needle manipulation. Once the De-qi sensation is achieved, the needle is retained for 30 min. Needle manipulation is performed every 10 min (by reinforcing the lifting-thrusting and twirling maneuvers of the needle). After 30 min, the needle is removed. All procedures must be carried out in strict accordance with the standard operating procedures.

**Exercise Therapy:** The use of elastic bands for resistance exercise, along with the exclusion of inappropriate exercise factors, forms the basis for guiding patients to undertake exercise therapy with Thera-band elastic bands. These elastic bands feature different resistance levels denoted by various colors. Teal, yellow, or red bands, which are suitable for the elderly, are selected. Each exercise session consists of a 10-minute warm-up and a 15 - to 30-minute resistance exercise phase [1,5,31].

**Exercise Movements:**

**Fig. 4:**
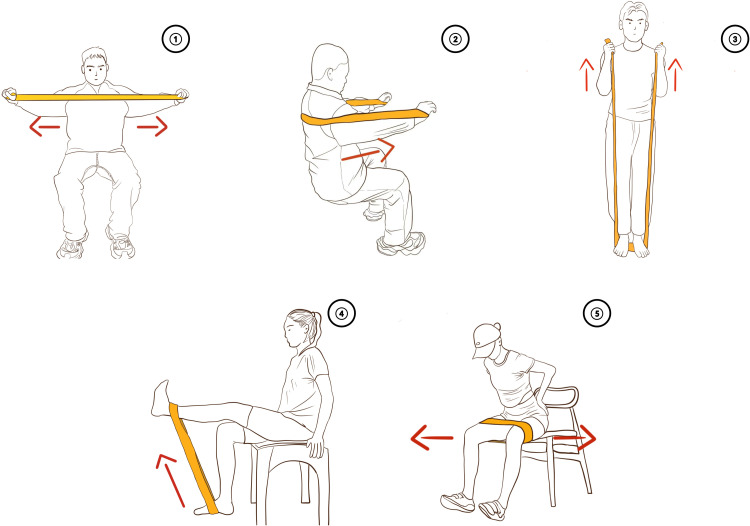
Illustration of exercise therapy movements.
(2) Seated Elastic Band Chest Push (Targeting the Triceps Brachii and Pectoralis Major). In ② of Fig. 4, the patient is seated. With the elastic band looped around the upper back and held by both hands, the upper limbs execute forward thrusting motions while countering the resistance of the elastic band. Each set contains 10 repetitions. Three sets are performed in total, with a one-minute rest between sets.
(3) Seated Elastic Band Biceps Curls (Targeting the Biceps). In ③ of Fig. 4, the patient holds both ends of the elastic band with both hands while the doctor helps to fix the middle part of the elastic band under the patient's feet. Subsequently, the patient conducts elbow flexion movements against the resistance of the elastic band. One set has 10 repetitions. A total of three sets are completed, with a one-minute rest between sets.
(4) Seated Unilateral Stirrups (Targeting the Quadriceps and Gluteus Maximus). As depicted in ④ of Fig. 4, the patient takes a sitting position. The doctor helps the patient to fasten one end of the elastic band to the ground and loops the other end around the patient's ankle joint. Then, the patient performs knee flexion and extension movements against the resistance of the elastic band, alternating between the two lower limbs. Each set involves 10 repetitions. Three sets are carried out in total, with a one-minute rest between sets.
(5) Seated Hip External Rotation (Targeting the Deep External Rotator Muscles around the Hip and the Gluteus Maximus). In ⑤ of Fig. 4, the patient is seated. The doctor loops the elastic band around the middle and lower parts of the patient's lower thighs. The patient then executes leg opening and closing movements while resisting the resistance of the elastic band. One set consists of 10 repetitions. Three sets are performed in total, with a one-minute rest between sets.

(1) Seated Elastic Band Chest Expansion (Targeting the Triceps Brachii and Deltoid Muscles). As shown in ① of Fig. 4, the patient sits upright and grasps the ends of the elastic band placed in front of the chest with both hands. Against the resistance of the elastic band, the patient performs chest expansion movements. One set comprises 10 repetitions. A total of three sets are carried out, with a one-minute rest interval between each set.

All the above-mentioned movements should be carried out under the supervision and assistance of the doctor.

Time Design: Upper and lower extremity training is alternated. Each exercise session lasts for 15 to 30 min. Initially, one movement training for each upper and lower extremity is carried out. Subsequently, the intensity is gradually increased according to individual differences. Each exercise session starts 1 to 2 h after a meal and is conducted every other day.

The treatment is administered on alternate days for 60 days, followed by a 90-day follow-up period.

#### Sham acupuncture group treatment protocol

In this group, the combination of sham acupuncture [32,33] and exercise therapy will be adopted. Sham acupuncture is performed by superficially pricking with a 0.30 mm × 40 mm needle at a location 2 cm away from the lateral edge of each acupoint selected for the acupuncture group (non-meridian and non-acupoint sites). No needle manipulation is involved, and the needle is retained for 30 min.

The exercise therapy in this group is identical to that in the acupuncture group. Similarly, the treatment and follow-up schedules are the same as those of the acupuncture group.

## Criteria for discontinuing or modifying allocated interventions

(1) In case of disease progression or the occurrence or potential occurrence of serious adverse events, the patient should withdraw from the clinical study. Those with aggravated conditions will be treated as invalid cases;
(2) During the research process, if the subjects develop certain comorbidities, complications, or experience special physiological changes that may render them unfit to continue participating in the study, they should withdraw from the clinical study according to the doctor's judgment;
(3) If the subjects are not satisfied with the efficacy of acupuncture or exercise therapy during the clinical study and are unwilling to continue participating in the clinical study, and they submit a request to withdraw from the clinical study to the attending doctor, they can withdraw from the study;
(4) Subjects with poor compliance to acupuncture treatment, who violate the treatment protocol, or who receive other concurrent treatments that may affect the judgment of treatment efficacy.

### Strategies to improve adherence to interventions

First and foremost, the researchers from the three centers underwent rigorous training, enabling each of them to have a detailed understanding of all the study contents. The subjects participating in this research will receive free physical examinations, DXA examinations, QCT examinations, and relevant blood index examinations throughout the study. Considering that elderly patients with SP may have mobility issues, we will provide free pick-up and drop-off services for the subjects, subject to the approval of the ethics committee.

### Relevant concomitant care permitted or prohibited during the trial

Participants remain on their standard treatment and medication procedures throughout the study period, and clinicians are advised to manage participants in the usual manner subject to the caveats outlined above.

### Outcomes

#### Primary efficacy indicator

(1) The Appendicular Skeletal Muscle Mass: Both the Asian Working Group for Sarcopenia and the European Working Group on SP in Older People have mentioned that ASM measured by DXA is an important index for the diagnosis and efficacy evaluation of sarcopenia, which can directly reflect the changes in muscle mass [1,2]. A value of <7.0 kg/m^2^ in men and <5.4 kg/m^2^ in women is considered positive.
(2) Handgrip strength: Handgrip strength is an important indicator for measuring muscle strength and one of the diagnostic criteria for SP. We use an electronic digital grip dynamometer. The patient stands with the elbow extended, and the dominant hand performs an isometric contraction with maximum force. The measurement is taken twice on the same hand with an interval of at least 15 s, and the maximum reading (kg) is selected. By referring to the recommendations of the Asian Working Group for Sarcopenia, we set the threshold as <28 kg for men and <18 kg for women [1].

#### Secondary outcome indicators

(1) The abdominal muscle area and muscle density at the L3 level measured by QCT: It is the clinical reference standards for measuring skeletal muscle mass and cross-sectional area. Since there is no unified threshold for evaluating SP using this method, we regard it as a secondary efficacy indicator. In this study, the thresholds recommended by Carey et al. through a large multi-center study are as follows: SMI ≤ 39 cm²/m² for women and SMI ≤ 50 cm²/m² for men [34,35].
(2) 6-meter walking speed: Walking speed is an important indicator for observing the limb function of SP patients. It can reflect patients' motor ability and systemic function, and is closely related to adverse outcomes of SP such as disability, cognitive impairment, hospitalization needs, falls, and mortality. In this protocol, the 6-meter walking speed test is adopted: Subjects are required to walk at their usual walking speed. During the assessment, when the subject's toes touch the 0-meter mark line, the timing starts from the first step beyond the 0-meter line and ends when the first step beyond the 6-meter line is taken. The corresponding time (seconds) is recorded. The test is conducted three times in total, with a 1-minute rest between each test, and the minimum value is taken as the test indicator [1].
(3) FTSST: FTSST is also an indicator for observing patients' limb function. The subjects cross their hands in front of the chest, stand up from the chair until both lower limbs are fully extended, and then sit down. This is repeated continuously for 5 times, and the required time is calculated. The experiment is repeated 3 times, and the shortest time is recorded [1].
(4) SPPB: SPPB mainly includes the assessment of three parts, namely gait speed, balance test, and chair stand test. The maximum score is 12 points. When the score is ≤ 8 points, it indicates poor physical performance. In terms of the gait speed test, methods such as the 4-meter usual walking speed test are usually adopted. The speed can be obtained by manually measuring the gait time with a stopwatch or using electronic devices. The balance test and chair stand test also have their own standard test procedures and scoring rules. These tests comprehensively reflect the physical performance status of an individual [1].
(5) calf/waist/hip circumference: Calf/waist/hip circumference is an indirect indicator reflecting the muscle mass of patients and has certain value in observing the efficacy of SP. Using a non-elastic measuring tape, the circumferences are measured at the umbilical level, the most prominent part of the medial thigh muscles on the right side, and the maximum circumferences of both calves while the subject is standing and dressed [36,37].
(6) Srum irisin/Myostatin: Myostatin is an actin secreted by skeletal muscle. The elevation of its plasma level can increase skeletal muscle fibrosis and lead to muscle atrophy. It can reduce skeletal muscle mass by regulating the division cycle of myoblasts and the expression of myogenic regulatory factors, ultimately inducing SP [38,39].Furthermore, studies have indicated that Myostatin, together with LIF (Leukemia Inhibitory Factor), IL-6 (Interleukin-6), and IL-7 (Interleukin-7), jointly participates in muscle hypertrophy and myogenesis. This finding serves to demonstrate that Myostatin plays a role in the growth and development of skeletal muscle itself and exerts a significant impact on local muscle function [40]. Irisin can alleviate the negative impact of Myostatin on musculoskeletal metabolism. The two regulate each other to balance skeletal muscle mass [41,42].Irisin is dependent on PGC-1α (Peroxisome Proliferator-Activated Receptor γ Coactivator 1α), whose core function is to drive brown-fat-like development. The primary role of brown adipose tissue is to consume energy through thermogenesis; thus, Irisin may hold significant implications for energy metabolism, particularly in obesity-related metabolic regulation. This underscores the impact of skeletal muscle—as a secretory organ—on systemic metabolism [40]. Daniel de Luis et al. have indicated that Irisin levels were significantly lower in patients with sarcopenia than in non-sarcopenic individuals; furthermore, higher Irisin levels were associated with a 61 % reduction in the risk of sarcopenia, and Irisin levels showed a positive correlation with AMSI. In contrast, Myostatin levels were negatively correlated with muscle mass, and Myostatin levels were 2.3-fold higher in elderly patients with sarcopenia [43]. Collectively, through the regulation of energy metabolism, the balance of muscle anabolism/catabolism, and the bone-muscle crosstalk network, Irisin and Myostatin play a central role in sarcopenia. Therefore, we believe that serum irisin and serum Myostatin have the potential to be indicators of the efficacy of SP treatment and have included them in the indicators of this protocol. The levels of serum irisin and myostatin will be measured by the researchers using enzyme - linked immunosorbent assay (ELISA) kits.

In summary, the efficacy indicators included in our study can comprehensively observe the muscle mass, muscle strength, and limb function of the patients, Table 1 provides a detailed explanation of this.

**Table 1:** Table of efficacy indicators. (All abbreviations in the table are presented in "Abbreviations").

**No.**	**Indicators**	**Primary / Secondary**	**Method**	**Effect**	**Refs.**
1	ASM	Primary	DXA	Reflect the muscle mass	[1,2]
2	Handgrip strength	Primary	An electronic dial-type hand- held dynamometer is adopted	Reflect muscle strength	[1]
3	The abdominal muscle area and (1) density at the L3 level measured	Secondary	QCT	Reflect muscle mass and cross-sectional area	[34,35]
4	6-meter walking speed	Secondary	Timing of walking at an average gait speed	Reflect somatic function	[1]
5	FTSST	Secondary	Timing of the subjects' sit-to-stand movement	Reflect somatic function	[1]
6	SPPB	Secondary	Scale-based Examination	Reflect somatic function	[1]
7	calf/waist/hip circumference	Secondary	Measurement with a non-elastic tape measure	Indirectly reflect the muscle mass	[36,37]
8	Srum irisin/Myostatin	Secondary	Serum examination	Reflect the muscle growth from the perspectives of cellular and organismal metabolism	[17–25]

#### Safety indicators

General physical examination items consist of height, weight, and body mass index (BMI), among others. Laboratory tests involve routine blood tests, blood glucose level monitoring, liver and kidney function assessments, as well as electrocardiogram (ECG) examinations. Additionally, special attention will be paid to observing any possible adverse reactions. These may be classified into local skin reactions and systemic reactions. Local skin reactions might present as allergic or non-allergic dermatitis, characterized by symptoms such as papules, skin erythema, edema, pruritus, blisters, or desquamation. Local discomfort may manifest as soreness, numbness, distension, or pain. Moreover, local hemorrhage and bruising could occur. Systemic reactions may include injection sickness and other conditions.

### Participant timeline

**Table:** 

	**STUDY PERIOD**					
	**Enrolment**		**Post-allocation**		**Follow-up**	
TIMEPOINT**	-t_1_	0	t_1_	t_2_	t_3_	t_4_
**ENROLMENT:**						
**Eligibility screen**	X					
**Informed consent**	X					
**[information acquisition]**	X					
**Allocation**	X					
**INTERVENTIONS:**						
**[Acupuncture group(Acupuncture+Exercise)]**		↔				
**[Sham Acupuncture group(Sham Acupuncture+Exercise)]**		↔				
**ASSESSMENTS:**						
**[SARC-Calf]**	X		X	X	X	X
**[ASM]**	X			X		X
**[The abdominal muscle area and muscle density at the L3 level measured]**		X		X		X
**[Handgrip strength]**	X		X	X	X	X
**[6-meter walking speed]**	X		X	X	X	X
**[FTSST]**	X		X	X	X	X
**[SPPB]**	X		X	X	X	X
**[calf/waist/hip circumference]**		X	X	X	X	X
**[Srum irisin/Myostatin]**		X		X		X
**[routine blood test]**		X		X		X
**[blood glucose]**		X		X		X
**[hepatorenal function tests]**		X		X		X
**[ECG]**		X		X		X

**Specific Timepoints:**t_1_** represents the stage of recruitment and diagnostic screening; **0** represents the baseline; **t_1_** represents the Visit 1 (30 days after treatment); **t_2_** represents the Visit 2 (60 days after treatment); **t_3_** represents the Visit 3 (30 days after the end of treatment); **t_4_** represents the Visit 4 (60 days after the end of treatment).

(All abbreviations in the table are presented in "Abbreviations".)

### Data collection and management

#### Plans for assessment and collection of outcomes

The primary efficacy indicator are ASM and Handgrip strength. The secondary outcome indicators encompass the abdominal muscle area and muscle density at the level of the third lumbar vertebra, including the rectus abdominis, transverse abdominis, internal and external obliques, quadratus lumborum, psoas major, and paraspinal muscles, which are quantified via QCT. Also included are 6-meter walking speed, the score of the FTSST, the score of the SPPB, calf/waist/hip circumference, serum irisin/myostatin. Considering the radiation from imaging examinations and the pain caused to patients by blood tests, the frequency and time of observation for each indicator are not completely the same, as detailed in Table 2.

**Table 2:** Time schedule for data collection of each indicator. (All abbreviations in the table are presented in "Abbreviations").

**No.**	**Indicators**	**baseline**	**Visit 1 (30 days after treatment)**	**Visit 2 (60 days after treatment)**	**Visit 3 (30 days after the end of treatment)**	**Visit 4 (90 days after the end of treatment)**
**1**	SARC—Calf	**√**	**√**	**√**	**√**	**√**
**2**	ASM	**√**		**√**		**√**
**3**	The abdominal muscle area and muscle density at the L3 level measured	**√**		**√**		**√**
**4**	Handgrip strength	**√**	**√**	**√**	**√**	**√**
**5**	6-meter walking speed	**√**	**√**	**√**	**√**	**√**
**6**	FTSST	**√**	**√**	**√**	**√**	**√**
**7**	SPPB	**√**	**√**	**√**	**√**	**√**
**8**	calf/waist/hip circumference	**√**	**√**	**√**	**√**	**√**
**9**	Srum irisin/Myostatin	**√**		**√**		**√**
**10**	routine blood test	**√**		**√**		**√**
**11**	blood glucose	**√**		**√**		**√**
**12**	hepatorenal function tests	**√**		**√**		**√**
**13**	ECG	**√**		**√**		**√**

#### Plans to promote participant retention and complete follow-up

Prior to the commencement of the study, all researchers will undergo training and be allocated tasks rationally. Each enrolled subject will be assigned to a fixed researcher, who will maintain contact with the subject throughout the entire study process and be responsible for treatment and all examinations. Given that elderly patients with SP may experience mobility impairments, we will make every effort to understand each patient’s health status, mobility capacity, and the distance from their residence to the hospital before enrollment to ensure the completion rate. Regarding transportation, we will provide three options for patients: 1. With the approval of the Ethics Committee, we will offer shuttle services to patients; 2. For patients who do not require shuttle services, we will provide an appropriate transportation allowance; 3. For individual patients with severe mobility limitations, we will dispatch medical staff to their homes to conduct acupuncture treatment and exercise guidance, subject to the approval of the Ethics Committee. One week before each examination and assessment, researchers will contact the participants to ensure their attendance on schedule. If a participant fails to attend the examination and assessment as scheduled, we will reschedule within one week to allow them to complete the process.

#### Data management

Clinical data will be collected and recorded by trained research assistants at our research center. The clinical examination data will first be entered into case report forms and then electronically input. Another technician will conduct a consistency check to ensure the accuracy of data entry. All data will be stored in password-protected computers. This study will be conducted in accordance with the Good Clinical Practice (GCP) to safeguard the rights, interests, and well-being of the participants, and to ensure that the collected data are complete and verifiable from the source documents. Patients can withdraw from the study at any time without giving any reason, and their medical care or legal rights will not be affected. After the completion of the study, the patient files will be retained for three years.

#### Confidentiality

All records containing personal information will be stored separately in a database and shared solely for research purposes within the scope of this study. Only healthcare professionals and researchers have the right to access the information required to carry out the interventions.

#### Plans for collection, laboratory evaluation and storage of biological specimens for genetic or molecular analysis in this trial/future use

Blood samples will be collected at the three research centers for evaluation purposes. Once the samples reach the local research laboratories at each center, collection, transportation, storage, and preparation will be carried out in accordance with local protocols. Prior to being processed within the specified time, the blood samples need to be stored at a temperature range of 2 - 8 °C. All samples collected during the trial will be labeled with patient identification codes and will not contain any identifiable data. Patients have the option to consent to the retention of their samples for future research use. The samples will be stored anonymously in a central location. After the completion of the study, they will be preserved for a minimum of 5 years and a maximum of 10 years, and then these specimens will be destroyed by incineration according to local guidelines and protocols. The potential uses of the stored samples have been included in the informed consent form. However, any further use of the samples still requires the approval of the institutional ethics committee.

### Statistical methods

#### Statistical methods for primary and secondary outcomes

The main efficacy indicators will be analyzed using both the full analysis set (FAS) and per protocol set (PPS). The other efficacy indicators will be analyzed via the FAS set, while the safety indicators will be analyzed with the safety set (SS). The baseline equilibrium analysis will also be carried out using the FAS set.

Statistical analysis encompasses statistical description and statistical inference. For the statistical description of quantitative indicators, it includes mean, standard deviation, median, minimum value, and maximum value. Qualitative indicators are depicted by frequency tables and constituent ratios. Statistical inference involves interval estimation and hypothesis testing. For quantitative indicators, their two-sided 95 % confidence intervals (CIs) will be estimated. Each efficacy indicator will undergo bilateral testing with a significance level of α = 5 %. The null hypothesis posits that there is no statistically significant difference between the test group and the control group, whereas the alternative hypothesis contends that there is a statistically significant difference between the two groups. When *p* < 0.05, the null hypothesis will be rejected, and the difference between the two groups is deemed statistically significant.

Regarding measurement data, normality analysis is performed first. All data conforming to normal distribution are expressed as mean ± standard deviation. For comparison within the same group before and after treatment, the paired *t*-test is utilized, and for comparison between groups, the independent samples *t*-test is employed. For non-normally distributed measurement data, the rank sum test is adopted. For non-graded data in count data, the chi-square or non-parametric test is used. For graded data, the Ridit test is applied. In a bilateral test, a P value <0.05 is considered as a statistically significant difference.

#### Methods for additional analyses

Prior to the protocol design, we conducted a systematic review and meta-analysis in this field (neither the paper was written nor published). This meta-analysis has been registered on the International Platform of Registered Systematic Review and Meta-analysis Protocols (INPLASY) (Registration number: INPLASY202480097). It served to obtain background information, identify the problems to be addressed in this study, assist in sample size estimation, and help us determine the outcome indicators.

#### Methods in analysis to handle protocol non-adherence and any statistical methods to handle missing data

We will conduct both intention-to-treat (ITT) analysis and per-protocol (PP) analysis simultaneously. Specifically, when interpreting the intervention effects, the ITT analysis will be more favorably regarded as the gold-standard method. However, the results of both the ITT analysis and the PP analysis will be reported together for the purpose of mutual comparison. When performing the ITT analysis with incomplete data, the multiple imputation technique will be utilized to impute the missing data.

#### Plans to give access to the full protocol, participant level-data and statistical code

The datasets analyzed during the current study, along with the statistical codes, are available upon reasonable request from the corresponding author. This article contains the complete research protocol. For any details that need to be known and specific operational questions, please contact the first author or the corresponding author for communication.

## Oversight and monitoring

### Composition of the coordinating centre and trial steering committee

The Trial Steering Committee consists of three members from the sponsor, including the principal investigator and the coordinating investigator. The coordinating investigator will obtain daily support for the trial from the various investigators. The Trial Steering Committee will be informed of the progress of the study on a monthly basis.They will conduct quality control and monitoring throughout the entire research process. Specifically, they will examine and confirm the accuracy and completeness of the recording and reporting of all research data, as well as the proper and full completion of case report forms, ensuring consistency with the original data.

### Composition of the data monitoring committee, its role and reporting structure

The Data Monitoring Committee (DMC) is composed of data scientists and statisticians from Shaanxi University of Chinese Medicine. Their primary responsibility is to ensure the data quality of the study by reviewing the trial every two months. The DMC operates independently of the sponsor, with no conflict of interest. Upon request of the corresponding author, the committee charter is available for further review.

### Adverse event reporting and harms

To safeguard the life and health of patients, we have strictly defined adverse events and formulated a comprehensive response plan for the detection, severity assessment, treatment, reporting, causal judgment regarding the treatment or examination measures used in the study, as well as the observation and recording of adverse events.

### Frequency and plans for auditing trial conduct

The implementation of the trial will be reviewed by the Data Monitoring Committee (DMC) on a quarterly basis. The review process will be conducted independently of the researchers and the sponsor.

### Plans for communicating important protocol amendments to relevant parties

We currently have no plans to modify this protocol. However, in the event of any changes to the protocol, they will be submitted by the principal investigator and must obtain approval from the Direct Research Funding Committee and the Ethics Committee prior to implementation. In addition, the subjects will be notified.

### Dissemination plans

Upon completion of the study, we will write up the research findings as a paper and publish it in a peer-reviewed journal, and disseminate the results to medical professionals, the public, and other relevant groups as soon as possible. The funder will play no role in the publication decision and impose no restrictions.

## Discussion

The etiological factors of SP include aging, chronic diseases, malnutrition, and physical inactivity [1,2]. As muscle serves not only as a locomotor organ but also as a crucial metabolic organ, SP not only impairs limb function but also leads to various adverse consequences, such as exacerbating metabolic disorders, affecting disease prognosis, and reducing quality of life [44]. The mechanisms underlying acupuncture in the treatment of SP involve regulating the neuroendocrine system, exerting anti-inflammatory and antioxidant effects, and improving neuromuscular function. By needling specific acupoints, the function of neuroendocrine systems such as the hypothalamic-pituitary-adrenal axis can be modulated. Studies have demonstrated that needling acupoints like Zusanli (ST36) can upregulate the serum level of insulin-like IGF-1, which in turn activates muscle satellite cells, promotes muscle protein synthesis, and increases the cross-sectional area of muscle fibers. Additionally, acupuncture can regulate the levels of stress hormones, thereby reducing muscle catabolism and maintaining the stability of muscle mass [45]. Furthermore, acupuncture for SP treatment possesses advantages such as individualization and multi-target regulation. Due to potential differences in the etiology and pathogenesis of SP, patients may present with varied symptoms and signs. By comprehensively evaluating the patient's constitution and disease characteristics, physicians can selectively choose acupoints for needling, thereby achieving precise treatment and enhancing therapeutic efficacy [46].

The observation indicators set in our protocol are relatively more comprehensive. Additionally, sham controls and a multi-center design have been incorporated to enhance the reliability. Through this study, we aim to make the diagnosis of SP and the acupuncture treatment protocol more complete, accurate, standardized, scientific, easy to operate, and promoteable, so as to improve the quality of life of SP patients and alleviate their economic burden. However, there are still some limitations. The collected samples are all from patient cases in this region, so the research results may have certain geographical limitations. Due to the long follow-up period, it is difficult to maintain the compliance of the research subjects, and there is a high risk of attrition bias.

### Protocol validation

The clinical study corresponding to our protocol is still underway, with subject recruitment and intervention therapy currently in progress. As we have not yet reached the outcome assessment phase, data to validate the protocol have not been generated.

### Limitations

There are still some limitations. The collected samples are all from patient cases in this region, so the research results may have certain geographical limitations. Due to the long follow-up period, it is difficult to maintain the compliance of the research subjects, and there is a high risk of attrition bias.

## CRediT author statement

FY, W-xL, and Y-cL designed this study. The manuscript was written by Y-cL., Y-cL and YL contributed to complete the inclusion and follow-up of patients in the clinical trial protocol. FY and W-xL contributed to the management and analysis of clinical trial data. All authors read and approved the final manuscript.

## Declaration of competing interest

The authors declare that they have no known competing financial interests or personal relationships that could have appeared to influence the work reported in this paper.

## Data Availability

No data was used for the research described in the article.

## Appendix Supplementary materials

Image, application 1
